# Implementation of 7-1-7 for improving timeliness in outbreak detection and response in Thailand: A mixed-methods study on challenges in application and achievement of targets in 2024-25

**DOI:** 10.12688/wellcomeopenres.25087.1

**Published:** 2026-02-12

**Authors:** Peeriya Watakulsin, Charuttaporn Jitpeera, Pitiphon Promduangsi, Khanuengnij Yueayai, Suphanat Wongsanuphat, Seesai Yeesoonsang, Patchanee Plernprom, Chonlada Siri, Kanyanach Kuljirakul, Thitinan Thintha, Kannika Songkram, Kulchayada Charoensiwarak, Soawapak Hinjoy

**Affiliations:** 1 Division of Epidemiology, Department of Disease Control, Ministry of Public Health, Nonthaburi, 11000, Thailand; 2 Division of Communicable Diseases, Department of Disease Control, Ministry of Public Health, Nonthaburi, 11000, Thailand; 3 Office of Disease Prevention and Control Region 9, Department of Disease Control, Ministry of Public Health, Nakhon Ratchasima, 30000, Thailand; 4 Office of Disease Prevention and Control Region 2, Department of Disease Control, Ministry of Public Health, Phitsanulok, 65000, Thailand; 5 Office of the Senior Expert Committee, Department of Disease Control, Ministry of Public Health, Nonthaburi, 11000, Thailand

**Keywords:** Health system, Global health security, International Health Regulations, Operational research, Timeliness, Outbreak, Emerging infectious disease

## Abstract

**Background:**

Infectious disease outbreaks result in unfavourable outcomes when swift actions fail. The 7-1-7 target (detect within 7 days, notify within 1 day, respond within 7 days) was proposed to improve timeliness. Thailand piloted 7-1-7 in 2024 and scaled it up nationwide in 2025. This study aimed to quantify the 7-1-7 target achievement, identify bottlenecks and enablers, and describe challenges and solutions for applying the 7-1-7 metrics.

**Methods:**

A mixed-methods study was conducted with quantitative assessment of all events meeting the national health threat criteria during January 2024 and June 2025 for timeliness, bottlenecks and enablers for meeting the 7-1-7 target. A qualitative descriptive study with in-depth interviews of key stakeholders explored challenges and solutions for applying the 7-1-7 framework.

**Results:**

Of the 1,435 events assessed, 15% were dangerous communicable diseases and 3% occurred in hotels. Among all the events, 50% achieved the 7-1-7 target; 85%, 73%, and 83% were detected, notified and responded to in a timely manner, respectively. The median (interquartile range) duration for detection, notification and response were 0 (0–5) days, 0 (0–2) days and 2 (0–5) days, respectively. Among events that occurred in hotels, none met the 7-1-7 target. Dangerous communicable diseases had the highest notification (95%) and response performance (99%), despite lower timely detection (71%). Key bottlenecks included delayed care-seeking and weak coordination. Key stakeholders felt that the 7-1-7 metrics was useful, but the lack of standardised data sources, disease-specific guidelines and centralised data extraction made implementation difficult. Proposed solutions focused on streamlining and decentralizing the application of 7-1-7.

**Conclusion:**

It is appreciable that Thailand was able to apply the 7-1-7 framework and half of the events met the 7-1-7 target. However, to better the application of 7-1-7, future work should focus on strengthening data integration and formulating disease specific guidelines.

## Plain Language Summary

When infectious diseases spread, it's important to act fast to prevent them from getting worse. Thailand adopted a framework called ‘7-1-7’ to help respond to disease outbreaks more quickly - the goal is to detect an outbreak within 7 days, report it within 1 day, and complete early responses within 7 days. Through this study, we wanted to see how well the 7-1-7 framework worked in Thailand and understand what made it successful or difficult to use.

We looked at 1,435 disease outbreaks that happened in Thailand between January 2024 and June 2025. We measured how quickly health officials detected, reported, and responded to these outbreaks. We also identified challenges which delayed these processes, and we interviewed health workers to understand their experiences applying the tool.

We found that half of all disease outbreaks met the 7-1-7 target. The system worked better for some diseases when compared to others - dangerous diseases were responded to faster than vaccine-preventable diseases like measles. Most outbreaks happened in communities. The biggest problems identified were people seeking medical care late, poor community awareness regarding diseases, and poor coordination between health organizations. Health workers found the 7-1-7 framework helpful, but faced the following challenges: there was no systematic way of collecting information, it increased their job burden, and they had difficulties in entering some of the data into the platform.

Using the 7-1-7 framework, we find that Thailand's health system responded quickly to disease outbreaks in the past year and a half. Improvements which can further improve timeliness include educating communities on when to seek care, and improving cooperation between health organizations. One of the shortcomings of this study was that data of events were incomplete, and the actions taken to overcome the problems identified were not documented.

## Introduction

Infectious disease outbreaks occur frequently and can result in high levels of morbidity and mortality, and strain healthcare systems if not dealt with in a timely fashion. There are numerous examples of how delayed detection, notification and response to outbreaks led to greater disease spread and additional lives lost ^1^. The Ebola outbreak in 2013 was misdiagnosed for months in Western African countries, and even after the disease was identified, coordinated responses were not initiated immediately. This led to the transmission of Ebola to neighbouring countries, and the loss of more than 11,000 lives ^1, 2^. The Covid-19 pandemic was a global disaster that lasted for around 3 years, which may have been better contained with timely outbreak detection, notification and response ^3^.

Because of these past experiences, the importance of measuring timeliness, and identifying gaps in outbreak preparedness and response is being recognised internationally. In 2021, Frieden et al proposed a new global target called 7-1-7, which aims for every suspected outbreak to be detected within seven days of emergence, notified within one day of detection and for early responses to be completed within seven days of notification ^4^. Meeting this 7-1-7 criteria is a new target included in the 14 ^th^ General Programme of Work published by the World Health Organization (WHO) in 2025, and the Pandemic Fund’s results framework now includes the number of countries that have applied the 7-1-7 approach as one of the core indicators on capability ^5, 6^. To increase the achievement of the 7-1-7 target, WHO encourages all countries to perform early action reviews (EARs) for public health events, and has released a guidance document to facilitate the same. EARs are a rapid, performance-improvement exercise that evaluates how quickly and effectively early detection, notification, and response activities are carried out during health events, fostering continuous learning and collaboration among stakeholders ^7^.

In 2023, a study was published which assessed the feasibility and utility of the 7-1-7 approach in evaluating health system performance in detecting, notifying and responding to outbreaks in five countries. It was found that the 7-1-7 target provided a useful framework which enabled countries to assess their own outbreak preparedness; bottlenecks and enablers captured were useful in prioritising activities to improve timeliness. In those five countries, 27% of events met all the three targets ^8^. Since then, the uptake of this framework has increased globally and to date, around 20 countries are included in the 7-1-7 Alliance, which is created and hosted by Resolve to Save Lives (RTSL) ^9^.

In Thailand, in January 2024, a team of subnational government officials working in Health Region 2 grew interested in the concept and began piloting the 7-1-7 tool in the area. Using the material available on the 7-1-7 Alliance website, they learnt how to adopt the framework, and then trained staff working in the Epidemiology Unit to apply the metrics to retrospective events reported to the Department of Disease Control (DDC). The report generated from this pilot enabled Health Region 2 to have baseline timeliness information on detection, notification, and early responses for each disease, in different settings. This facilitated knowledge exchange with other health regions and countries. The findings demonstrated gaps in detection, particularly for vaccine-preventable diseases. The utilization of the tool also improved outbreak preparedness in Health Region 2. For example, there was a delay in the detection of a diarrhoea outbreak in a camp. The bottleneck causing this was found to be non-governmental organisation (NGO) staff working in the camp using wrong channels to communicate with the local public health staff. This bottleneck was reported to higher level authorities and protocols for data sharing were implemented promptly to improve communication and enable timely detection ^10^.

In 2025, with the support of DDC's “Prevent – Detect – Response” policy, government officials recognized the importance and opportunity to upscale the adoption of the 7-1-7 framework to the entire country. Staff in all health regions were trained in January 2025, and they applied the metrics to all events recorded from January 2024 onwards. This provided us the opportunity to assess Thailand’s outbreak preparedness and response between January 2024 and June 2025. By identifying bottlenecks in applying the framework and meeting its targets, efforts can be put towards overcoming these and accelerating the processes of detection, notification and response. Accordingly, this study was conducted to 1. determine the proportion of events that achieved the 7-1-7 target, as well as each component of it; 2. identify the bottlenecks and enablers in achieving the 7-1-7 target, as reported on the online database; and 3. describe the facilitators, challenges and solutions for applying the 7-1-7 framework at the national and regional levels.

## Methods

### Study design

We conducted a mixed-methods study, which comprised quantitative and qualitative components. The quantitative component was a descriptive longitudinal study using programmatic data, and the qualitative component was a descriptive study using in-depth interviews.

### Setting

***General setting.*** Thailand, with a population of over 70 million, is a regional hub for travel and trade in Southeast Asia. Its healthcare system, governed by the Ministry of Public Health (MoPH), is structured into five levels: national, regional, provincial, district, and subdistrict. At the national level, the MoPH oversees policy formulation, strategic planning, and resource allocation. However, approximately half of the subdistrict health facilities are operated by local government organizations under the Ministry of Interior, not the MoPH.

**Figure 1.**
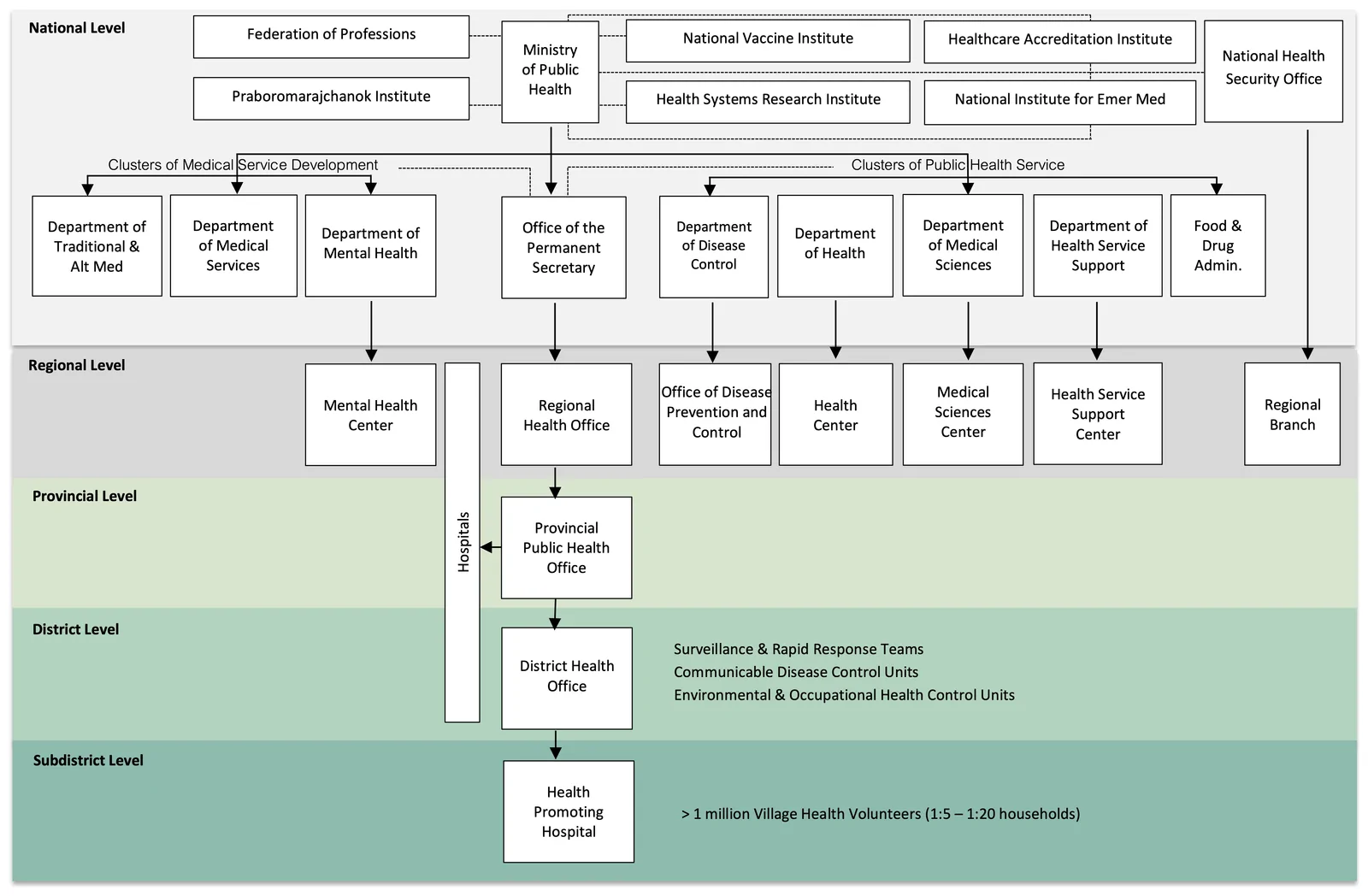
Structure of Health Facilities under the Thai Ministry of Public Health.

The regional level ensures coordination among provinces, by providing technical and administrative support. At the provincial level, Provincial Health Offices (PHOs) oversee health services across districts, acting as a bridge between regional and district systems. District Health Offices (DHOs) are responsible for implementing public health programs and managing healthcare facilities within their jurisdictions. Finally, the subdistrict level, comprising subdistrict health promoting hospitals and village health volunteers, provides essential health services and serves as the first line of service delivery ( Figure 1).

Thailand is divided into 13 health regions based on geographical distribution. Health Region 2 was the first area to pilot the 7-1-7 framework. This region, situated in the lower northern part of the country, covers areas along the Thai-Myanmar border where refugee camps are located, which creates unique challenges for disease control. This region faces complex health issues, including cross-border transmission of diseases, limited access to healthcare among migrant populations, and the constant threat of emerging diseases and large-scale outbreaks.

Thailand’s geographically diverse landscape, ranging from urban megacities like Bangkok to remote rural areas, creates variations in healthcare delivery and accessibility. Nevertheless, the country's commitment to universal health coverage (UHC) has improved health outcomes and access to care. Public health initiatives are further supported by Thailand’s extensive network of village health volunteers, who play a critical role in community-based surveillance and health promotion.

**Figure 2.**
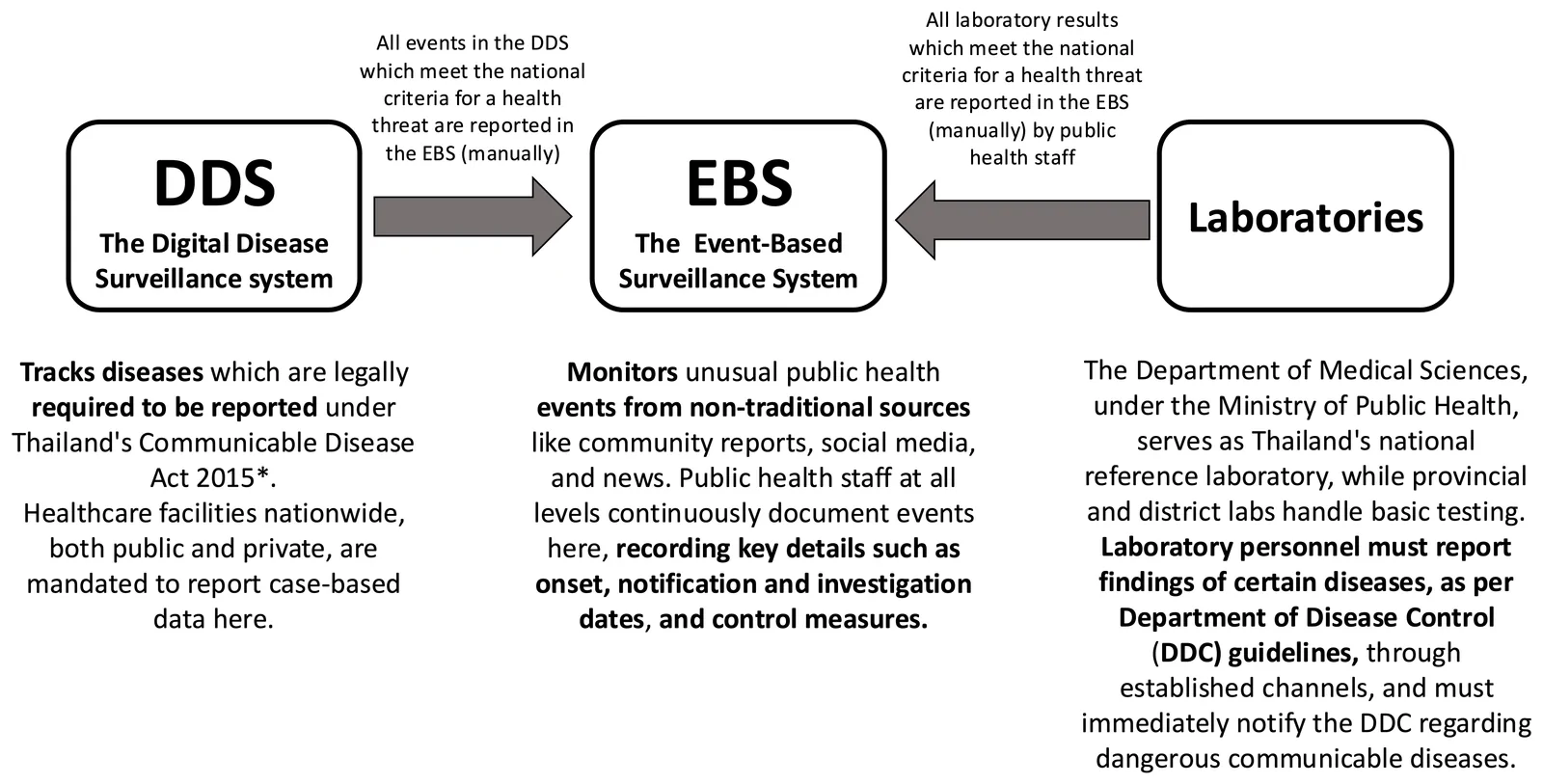
Disease Surveillance Systems in Thailand as of 2025.

***Surveillance system.*** Thailand's disease surveillance systems are designed to monitor, prevent, and control both communicable and non-communicable diseases. These systems operate under the DDC in the MoPH, which is designated to ensure timely detection and response to public health threats. There are three primary surveillance systems through which all diseases are notified to the DDC: the indicator based Digital Disease Surveillance System (DDS), the Event-Based Surveillance System (EBS), and Laboratory Services ( Figure 2). The Offices of Disease Prevention and Control in all 13 health regions report data on all health events that meet the national criteria for health threats (Supplementary 1) to the DDC via the EBS. Following verification of an event and the confirmation that public health interventions are required, the outbreak response system is promptly activated to coordinate and implement appropriate control measures. Collectively, these components constitute an integral early warning system designed to facilitate the rapid detection and response to disease outbreaks and other public health threats.

Applying timeliness metrics is mandated under The Thai Communicable Diseases Act, 2015 for 13 dangerous communicable diseases (DCDs) and 57 communicable diseases under surveillance. DCDs are highly virulent communicable diseases which are rapidly transmissible to other persons. They include Plague, Ebola Virus Disease, Smallpox, Yellow Fever, Severe Acute Respiratory Syndrome (SARS), Middle East Respiratory Syndrome (MERS), Marburg Virus Disease, Hendra Virus Infection, Nipah Virus Infection, Lassa Fever, Crimean-Congo Haemorrhagic Fever, West Nile Fever, and Extensively Drug-Resistant Tuberculosis (XDR-TB). These diseases are supposed to be reported within 3 hours and investigated within 12 hours. Communicable diseases under surveillance are required to be notified within 7 days and the timeliness metric for investigation depends on the disease type ^11^.

***Outbreak response system.*** Under the International Health Regulations (IHR) framework, Thailand has established Surveillance and Rapid Response Teams (SRRT) and Communicable Disease Control Unit teams (CDCU), depending on the level of response, which function from the national level to the sub-district level. These teams are responsible for detecting outbreak signals, investigating outbreaks, and implementing control measures.

***7-1-7 adoption in Thailand.*** Thailand's adoption of the 7-1-7 framework began in 2024 in Health Region 2, with a pilot retrospective analysis of data from January 1, 2019, to December 31, 2023. To scale adoption up to all regions, in early 2025, a dedicated 7-1-7 working group was established. They translated resource materials to Thai, established an e-learning platform, and developed a new database which was partially integrated with the existing EBS, to document data to assess the 7-1-7 timeliness metrics. Multiple workshops were conducted to train staff from the DDC on the metrics. To further enhance its sustainability, the 7-1-7 approach was incorporated into intermediate- to advanced-level epidemiology training, including the Field Epidemiology Training Program (FETP) and the Field Epidemiology Management Training (FEMT) curricula.

As part of the adoption of 7-1-7, After Action Reviews (AAR) were initiated in Thailand. These reviews take place after an outbreak investigation is closed, and are usually attended by the stakeholders involved in the response, including rapid response teams, related government staff, and NGOs. The feedback received in AARs guides further actions to be taken to improve outbreak preparedness. Furthermore, the country's IHR focal points were engaged to identify specific areas within Thailand's National Action Plan for Health Security (NAPHS) that could be strengthened based on evidence from the 7-1-7 assessment.

To enhance One Health integration in the adoption of 7-1-7 in Thailand and to address zoonotic disease surveillance and response, the Department of Livestock Development was partnered with. The 7-1-7 tools and methodologies were adapted to incorporate veterinary surveillance systems and environmental monitoring protocols. This multi-sectoral approach for 7-1-7 was endorsed during a high-level One Health meeting convened in August 2025.

***Information management system for 7-1-7 in Thailand.*** The 7-1-7 metrics are applied to all health events that meet the national criteria for a health threat. Data on these events relevant to the 7-1-7 target are entered into and managed through a newly created relational database using PostgreSQL, called EBS-717, which is partially integrated with the existing EBS. This database management system was chosen for its capacity to handle complex operations and its comprehensive security architecture.

DDC staff, primarily public health officers with rapid response experience, at the regional level are responsible for entering data of all events which occur in their region into the platform. They had previously attended a comprehensive three-day training workshop on 7-1-7. On entering the event ID into EBS-717, disease related characteristics auto-populate. However, the rest of the data, including key dates, bottlenecks in meeting detection, notification and response targets (as free text and categorised as per the bottleneck taxonomy ^12^), and enablers (as free text and categorised as mirror images of the bottlenecks) have to be entered manually. The staff sourced this data by systematically reviewing multiple sources including the EBS, spot reports, outbreak investigation reports and laboratory test results.

To ensure data quality and accuracy, the EBS-717 has incorporated defined data boundaries for each variable to minimize entry errors. Additionally, physicians and public health professionals with advanced training in field epidemiology have been designated as regional consultants, each serving in 1 or 2 health regions. They were provided with a three-day onsite training on 7-1-7. Their responsibilities include providing guidance when questions arise and randomly checking the data entered for completeness and accuracy of at least 25% of all events. The 25% cutoff was chosen to avoid over-burdening them, since they had to undertake these responsibilities in addition to their routine work. However, for this study, all 8 consultants checked the data quality of all the events (100%).

***Evaluating the 7-1-7 metrics for the current project.*** The 7-1-7 framework for measuring timeliness in outbreak detection, notification, and response is described in ( Table 1). To evaluate the 7-1-7 timeliness metrics for health events in Thailand, data was extracted from EBS-717, the newly created relational database.

**Table 1.** Definitions of 7-1-7 components and targets.

7-1-7 Component	Definition & Process	Target Timeline
**Detection** **(First-7)**	The number of days between **emergence** and **detection** of an outbreak/event. ➢ Emergence: • *Endemic*: date when number of cases increases above baseline. • *Non-endemic*: date when the index/linked case develops symptoms. • *Other events*: date when the event meets the reportable criteria. ➢ Detection = date when the event is first recorded in any system (paper, electronic, mobile).	**Within 7 days** of emergence
**Notification** **(Next-1)**	The number of days between **detection** and **notification** of an outbreak/event. Notification: date when an event is notified to appropriate public health authorities, usually via the established reporting channels.	**Within 1 day** of detection
**Early Response** **Actions** **(Second-7)**	The number of days between **notification** and **completion of early response actions** for an outbreak/event. Seven early response actions to be implemented: 1. Investigations & team deployment 2. Epidemiological analysis & risk assessment 3. Laboratory confirmation 4. Case management & IPC in facilities 5. Public health countermeasures (e.g., vaccines, ORS, PPE) 6. Risk communication & community engagement 7. Coordination mechanism ➢ Early response actions • *Initiation*: date first action occurs • *Completion*: date all applicable actions are completed	**Within 7 days** of notification

### Study population

For objective 1 and 2, all public health events that met the national criteria for a health threat recorded in DDC's EBS between January 1, 2024 and June 30, 2025 were assessed for this study. Events were excluded if they were duplicate records or involved non-communicable conditions such as injuries, disasters, occupational and environmental diseases. All events to which the 7-1-7 framework could be applied, based on the availability of key dates, were included in the timeliness analysis. The 7-1-7 framework could be applied to an event which had at least one of the following indicators available: number of days between emergence and detection, number of days between detection and notification, number of days between notification and response.

For objective 3, we interviewed directors and deputy directors (executives) from different health regions who had attended the second day of the 7-1-7 framework high-level meeting in August 2025. Additionally, we conducted online interviews with one public health officer from each region. These officers had been trained, and served as focal points of contact, responsible for the adoption of 7-1-7 in their region, and had at least six months of experience with public health surveillance or emergency response. Public health officers were also included from regions with the highest numbers of recorded events. In all, 6 interviews were conducted among executives, and 7 among public health officers, after which data saturation was achieved.

### Data sources and data variables

***Objectives 1 and 2.*** The total number of events between January 2024 and June 2025 were extracted from DDC's EBS. To apply the 7-1-7 framework, for all the eligible events, investigators extracted the following data from EBS-717’s database: 1) general event information including disease/disease group, geographic location, number of patients, number of deaths, event type, and setting; 2) key milestone dates (date of emergence, date of detection, date of notification, date of early response initiation, date of early response completion, and date of completion of each of the seven responses); 3) bottlenecks and enablers

***Objective 3.*** The facilitators, challenges and solutions for applying the 7-1-7 framework were determined by interviewing key stakeholders in their local language (Thai), using a semi-structured questionnaire with probes (Supplementary 2). Interviewers trained in qualitative research conducted the in-depth interviews to determine perceived facilitators and challenges in applying the 7-1-7 framework, along with possible solutions to enable its scale-up (Supplementary 3–5). Study participants were contacted at least a week in advance to schedule the interview at a convenient date and time, either in person or virtually. Interviews were audio recorded after taking informed consent (Supplementary 6), and field notes were also made. Transcripts were initially prepared in Thai and later translated to English.

### Data analysis, statistics and reporting

***Objective 1 and 2.*** We calculated the overall timeliness performance using R software, version 4.4.3 (R Core Team, 2025 by the R Foundation for Statistical Computing, Vienna, Austria) ^12^. We reported the median (and interquartile range) number of days taken to detect, notify and respond to an outbreak. Additionally, we reported the number and proportion of health events achieving the 7-1-7 target, as well as each component of it (timely detection in 7 days, notification in 1 day and early response in 7 days). Timeliness performances have also been reported for events categorised by disease clusters, settings and diseases. The number and proportion of identified bottlenecks extracted from the database have been reported, categorised as per the 7-1-7 Alliance’s bottleneck taxonomy ^12^. No such taxonomy exists for enablers, so a mirror image of the categories were created, and the number and proportion of enablers falling in each category have been reported.

***Objective 3.*** The qualitative transcripts were analysed using manual content analysis with inductive and deductive coding by two investigators. Themes related to facilitators, challenges and solutions in applying the 7-1-7 metrics at the national and regional level were identified. A third investigator reviewed the analysis and decisions on coding, and theme generation were made by consensus. The study has been reported as per STROBE ^13^.

### Statement on patient and public involvement

Patients and the public were not involved in the planning or conduct of this study.

### Ethical approval and consent to participate

The study was conducted according to the guidelines of the Declaration of Helsinki. Ethical approval was granted by the independent Ethical Review Committee of the Institute for the Development of Human Research Protections (IHRP), Thailand (approval number: IHRP No.45-2568, dated 28 April 2025). Written informed consent was obtained from all participants before interviews. No identifying personal data has been reported.

## Results

### Timeliness performance

**Figure 3.**
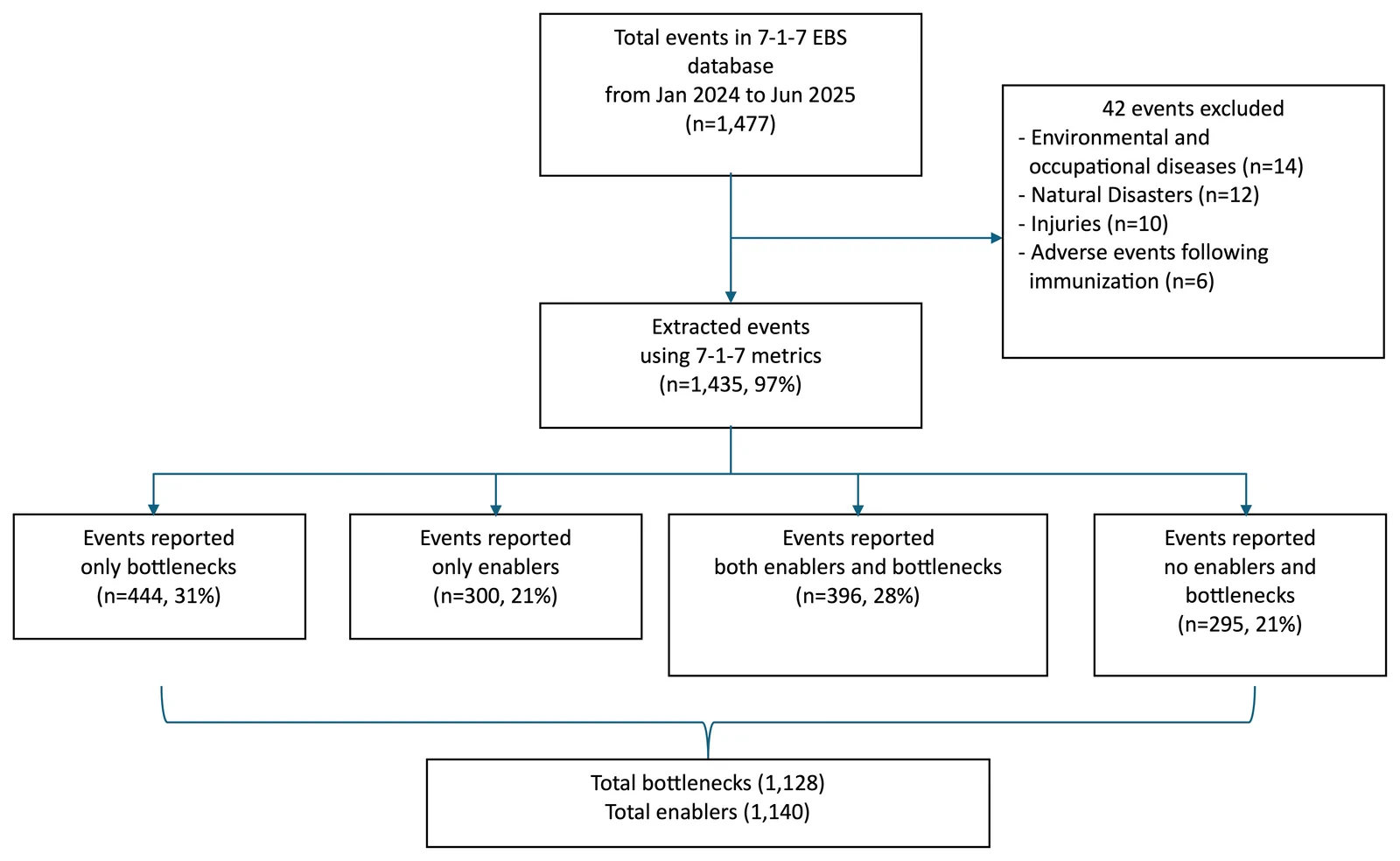
Data flow of all events from January 2024 to June 2025.

We included 1,435 public health events that occurred in the 13 regions of Thailand between January 2024 and June 2025. Out of them, 58% had complete data for all the 7-1-7 components ( Figure 3). Of these events, 950 events (66%) were events of individual cases, while 485 events (33%) involved multiple cases. Among all of the events, 494 (34%) had at least one reported death. The events were distributed across diverse settings, with community settings accounting for the largest proportion (1,042 events, 73%), followed by educational institutions (149 events, 10%) and prisons (73 events, 5%). Most events were respiratory tract infections (348 events, 24%), followed by DCDs or emerging infectious diseases (EIDs) (221 events, 15%), vector-borne diseases (195 events, 14%), and vaccine-preventable diseases (190 events, 13%).

Data availability for analysis varied across the three components of the 7-1-7 framework, with detection analysis possible in 1,299 events (91%), notification analysis in 1,241 (86%), and response analysis in 860 events (60%) ( Table 2). The overall performance of Thailand's public health system against the 7-1-7 target was: 410 events (50%) achieved the 7-1-7 target; 1,109 events (85%) were detected within 7 days, with a median detection time of 0 (IQR: 0 - 5) days (less than 24 hours); 903 events (73%) were notified within one day of detection, with a median notification time of 1 (IQR: 0-2) days; and in 718 events (83%), early response was completed within 7 days, with a median response time of 2 (IQR: 0-5) day.

**Table 2.** Timeliness and proportion achieving 7-1-7 targets by event settings, 1 Jan 2024 − 31 June 2025.

Setting	Events n	Events available for detection analysis n (%) *	Detection (target=7 days)		Events available for notification analysis n (%) *	Notification (target=1 day)		Events available for response analysis n (%) *	Completed Early Response (target= 7 days)		Events available for all analysis n (%) *	Events met all targets n (%) &
			Median (IQR)	Met target, n (%) #		Median (IQR)	Met target, n (%) $		Median (IQR)	Met target, n (%) ^		
Total	1435	1299 (91)	0 (0-5)	1109 (85)	1241 (86)	0 (0-2)	903 (73)	860 (60)	2 (0-5)	718 (83)	827 (58)	410 (50)
Community	1042	950 (91)	1 (0-5)	797 (84)	908 (87)	0 (0-2)	654 (72)	634 (61)	1 (0-4)	543 (86)	610 (59)	295 (48)
Educational Institution	149	136 (91)	0 (0-1)	131 (96)	132 (89)	0 (0-1)	108 (82)	89 (60)	4 (2-8)	66 (74)	87 (58)	46 (53)
Prison	73	66 (90)	0 (0-1)	65 (98)	65 (89)	0 (0-2)	48 (74)	54 (74)	5 (2-7)	41 (76)	51 (70)	32 (63)
Healthcare facility	60	55 (92)	2 (0-7)	42 (76)	53 (88)	0 (0-3)	37 (70)	29 (48)	1 (0-2)	27 (93)	29 (48)	16 (55)
Hotel	39	29 (74)	0 (0-26)	16 (55)	24 (62)	0 (0-1)	18 (75)	8 (21)	12 (8-18)	2 (25)	7 (18)	1 (14)
Military	25	21 (84)	0 (0-0)	20 (95)	20 (80)	0 (0-2)	13 (65)	17 (68)	3 (2-4)	15 (88)	14 (56)	8 (57)
Religious	12	12 (100)	0 (0-0)	12 (100)	12 (100)	0 (0-3)	8 (67)	10 (83)	2 (0-8)	7 (70)	10 (83)	5 (50)
Other	29	26 (90)	0 (0-2)	24 (92)	24 (83)	1 (0-4)	15 (63)	16 (55)	1 (1-6)	14 (88)	16 (55)	7 (44)
Unknown	6	4 (67)	7 (4-9)	2 (50)	3 (50)	0 (0-2)	2 (67)	3 (50)	1 (1-3)	3 (100)	3 (50)	0 (0)

* Percentage calculated with number of events as the denominator

# Percentage calculated with Number of Events with date of emergence and date of detection available as denominator

$ Percentage calculated with Number of Events with date of detection and notification available as denominator

^ Percentage calculated with Number of Events with date of notification and response available as denominator

& Percentage calculated with Number of Events with date of emergence, detection, notification and response available as denominator; 0 means less than 1 day

Notably, hotel settings showed the lowest data completeness across all the 7-1-7 components (29 events, 74% for detection; 24 events, 62% for notification; and 8 events, 21% for response), while healthcare facilities demonstrated particularly limited response data availability (29 events, 48%). Meanwhile, setting-specific analysis revealed substantial performance variations ( Table 2). Community settings, where majority of the events took place, achieved (797 events) 84% timely detection, (654 events) 72% timely notification and (543 events) 86% timely response. All events in religious settings were detected within 7 days (12 events, 100%), and most of them were respiratory infections, like influenza and Covid-19. Hotels performed poorly across all metrics, with zero events meeting the seven-day response target.

When examining performance by disease cluster, vaccine-preventable diseases and central nervous system infections had the lowest detection data availability (163 events, 86% and 11 events, 79% respectively), and central nervous system infections showed the poorest response data completeness (1 events, 7%). Almost all food- and water-borne diseases were detected within seven days, but only 63 events (73%) were responded to within the target Besides those involving the central nervous system, DCDs and EIDs showed the highest timely notification (194 events, 91%) and response performance (151 events, 96%), despite only 73% timely detection (156 events). Those of DCDs and EIDs which were reported later than the one-day target included 10 cases of XDR-TB, 19 suspected MERS cases, and a case of suspected Lassa fever. All the MERS and Lassa fever suspects had negative laboratory confirmatory tests. Contact-borne diseases performed poorly across notification (51%,) and response timeliness (77%) ( Table 3). The list of diseases included under each cluster, along with the performance of each individual disease has been included in Supplementary ( Table 1, Supplementary 7, Additional data).

**Table 3.** Timeliness and proportion achieving 7-1-7 targets by disease clusters, 1 Jan 2024 − 31 June 2025.

Disease Cluster	Events n	Events available for detection analysis n (%) *	Detection (target=7 days)		Events available for notification analysis n (%) *	Notification (target=1 day)		Events available for response analysis n (%) *	Completed Early Response (target= 7 days)		Events available for all analysis n (%) *	Events met all targets n (%) $
			Median (IQR)	Met target, n (%) #		Median (IQR)	Met target, n (%) $		Median (IQR)	Met target, n (%) ^		
Total	1435	1299 (91)	0 (0-5)	1109 (85)	1241 (86)	0 (0-2)	903 (73)	860 (60)	2 (0-5)	718 (83)	827 (58)	410 (50)
Respiratory tract infections	348	311 (89)	0 (0-3)	271 (87)	296 (85)	1 (0-5)	178 (60)	195 (56)	3 (0-6)	160 (82)	184 (53)	82 (45)
Dangerous / emerging diseases	221	215 (97)	5 (2-9)	153 (71)	211 (95)	0 (0-1)	189 (90)	201 (91)	1 (0-1)	198 (99)	200 (90)	126 (63)
Vector-borne diseases	195	181 (93)	0 (0-3)	164 (91)	173 (89)	0 (0-2)	116 (67)	98 (50)	4 (1-8)	71 (72)	93 (48)	39 (42)
Vaccine-preventable diseases	190	163 (86)	0 (0-6)	136 (83)	159 (84)	0 (0-2)	119 (75)	95 (50)	2 (1-5)	82 (86)	92 (48)	41 (45)
Zoonotic diseases	166	147 (89)	1 (0-5)	123 (84)	139 (84)	0 (0-1)	115 (83)	102 (61)	2 (1-6)	80 (78)	99 (60)	53 (54)
Food- and water-borne diseases	156	143 (92)	0 (0-1)	138 (97)	138 (88)	0 (0-1)	119 (86)	86 (55)	4 (2-8)	63 (73)	84 (54)	47 (56)
Contact-borne diseases	144	127 (88)	0 (0-1)	114 (90)	114 (79)	1 (0-5)	58 (51)	81 (56)	1 (0-7)	62 (77)	73 (51)	21 (29)
Central nervous system infections	14	11 (79)	2 (1,6)	9 (82)	10 (71)	0 (0-1)	9 (90)	1 (7)	1 (-)	1 (100)	1 (7)	1 (100)
Sexually transmitted diseases	1	1 (100)	0 (-)	1 (100)	1 (100)	35 (-)	0 (0)	1 (100)	3 (-)	1 (100)	1 (100)	0 (0)

* Percentage calculated with number of events as the denominator

# Percentage calculated with Number of Events with date of emergence and date of detection available as denominator

$ Percentage calculated with Number of Events with date of detection and notification available as denominator

^ Percentage calculated with Number of Events with date of notification and response available as denominator

& Percentage calculated with Number of Events with date of emergence, detection, notification and response available as denominator; 0 means less than 1 day

### Bottlenecks and enablers in meeting the 7-1-7 target

Of the 1,435 total events, at least one bottleneck or enabler was reported in 1,140 events (79%), with 840 events (59%) experiencing bottlenecks and 696 (49%) experiencing enablers ( Table 3). Bottlenecks in detection affected 530 events, followed by bottlenecks in response, which affected 259 events, and bottlenecks in notification, which were reported for 227 events. Patient and community factors emerged as the primary bottleneck theme (370 bottlenecks, 33% of all events that reported at least one bottleneck or enabler), predominantly characterized by delays in care-seeking behaviour (231 bottlenecks, 20%). Coordination challenges represented the second largest barrier (186 bottlenecks, 16%), with inadequate coordination across public health units being the most frequent issue (112 bottlenecks, 10%). Clinical and healthcare worker-related bottlenecks (172 bottlenecks, 15%) primarily consisted of low clinical awareness (86 bottlenecks, 8%) and inadequate surveillance training (78 bottlenecks, 7%) ( Table 4).

**Table 4.** Reported bottlenecks of achieving the 7-1-7 target in Thailand, 1 Jan 2024 − 31 June 2025.

Themes and Categories of Bottleneck	Detect *	Notify *	Response *	Total *
	N (%)	N (%)	N (%)	N (%)
**Patient & Community**	**313 (27.5)**	**20 (1.8)**	**37 (3.2)**	**370 (32.5)**
Delay in care-seeking by patient	227 (19.9)	0 (0)	4 (0.4)	231 (20.3)
Low community knowledge or trust	53 (4.6)	15 (1.3)	16 (1.4)	84 (7.4)
Inadequate sensitivity of community detection	24 (2.1)	2 (0.2)	0 (0)	26 (2.3)
Others	9 (0.8)	1 (0.1)	5 (0.4)	15 (1.3)
Inadequate risk communications or community engagement	0 (0)	2 (0.2)	12 (1.1)	14 (1.2)
**Coordination**	**31 (2.7)**	**96 (8.4)**	**59 (5.2)**	**186 (16.3)**
Inadequate coordination across public health units or agencies	12 (1.1)	72 (6.3)	28 (2.5)	112 (9.8)
Weak response coordination, including incident management and rapid response team capacity	7 (0.6)	1 (0.1)	20 (1.8)	28 (2.5)
Inadequate coordination with neighboring countries	2 (0.2)	10 (0.9)	1 (0.1)	13 (1.1)
Inadequate multisectoral/disciplinary response teams	3 (0.3)	5 (0.4)	5 (0.4)	13 (1.1)
Inadequate One Health information sharing/collaboration	1 (0.1)	2 (0.2)	5 (0.4)	8 (0.7)
Inadequate coordination between public and private sectors	3 (0.3)	4 (0.4)	0 (0)	7 (0.6)
Others	3 (0.3)	2 (0.2)	0 (0)	5 (0.4)
**Clinical or Health Care Worker**	**99 (8.7)**	**16 (1.4)**	**57 (5)**	**172 (15.1)**
Low awareness or clinical suspicion by health workers	68 (6)	9 (0.8)	9 (0.8)	86 (7.5)
Health professional with inadequate training in surveillance and response	25 (2.2)	7 (0.6)	46 (4)	78 (6.8)
Limited clinical case management capacity	5 (0.4)	0 (0)	1 (0.1)	6 (0.5)
Inadequate clinical surveillance focal point / capacity	1 (0.1)	0 (0)	1 (0.1)	2 (0.2)
**Laboratory**	**64 (5.6)**	**27 (2.4)**	**55 (4.8)**	**146 (12.8)**
Inadequate laboratory diagnostic capacity	15 (1.3)	13 (1.1)	9 (0.8)	37 (3.2)
Delayed specimen transportation	14 (1.2)	1 (0.1)	16 (1.4)	31 (2.7)
Laboratory reporting failure or delay	14 (1.2)	5 (0.4)	10 (0.9)	29 (2.5)
Slow internal laboratory turnaround time	12 (1.1)	5 (0.4)	6 (0.5)	23 (2)
Others	4 (0.4)	2 (0.2)	7 (0.6)	13 (1.1)
Delayed specimen collection	2 (0.2)	0 (0)	5 (0.4)	7 (0.6)
Inadequate diagnostic commodities (lab reagents, RDTs, specimen collection kits)	3 (0.3)	1 (0.1)	2 (0.2)	6 (0.5)
**Data Systems**	**24 (2.1)**	**43 (3.8)**	**7 (0.6)**	**74 (6.5)**
Lack of timely or complete surveillance data	17 (1.5)	38 (3.3)	4 (0.4)	59 (5.2)
Technological challenge for electronic surveillance/reporting systems (e.g., network coverage)	4 (0.4)	5 (0.4)	2 (0.2)	11 (1)
Others	3 (0.3)	0 (0)	1 (0.1)	4 (0.4)
**Planning & Procedures**	**22 (1.9)**	**23 (2)**	**29 (2.5)**	**74 (6.5)**
Failure to follow event notification procedures	9 (0.8)	17 (1.5)	9 (0.8)	35 (3.1)
Inadequate surveillance or response policies and guidelines	7 (0.6)	4 (0.4)	5 (0.4)	16 (1.4)
Inadequate risk assessments, preparedness, or response plans	2 (0.2)	0 (0)	12 (1.1)	14 (1.2)
Inadequate procedures in place for event notification	2 (0.2)	1 (0.1)	1 (0.1)	4 (0.4)
Failure to follow initial risk assessment or event verification procedure	0 (0)	1 (0.1)	2 (0.2)	3 (0.3)
Others	2 (0.2)	0 (0)	0 (0)	2 (0.2)
**Event Characteristics**	**24 (2.1)**	**4 (0.4)**	**25 (2.2)**	**53 (4.6)**
Others	15 (1.3)	2 (0.2)	13 (1.1)	30 (2.6)
Access issues (e.g., remote, fragile, conflict settings, climate conditions)	5 (0.4)	1 (0.1)	12 (1.1)	18 (1.6)
New, unexpected, or deprioritized pathogen	4 (0.4)	1 (0.1)	0 (0)	5 (0.4)
**Resouces & Procurement**	**3 (0.3)**	**2 (0.2)**	**14 (1.2)**	**19 (1.7)**
Logistics and shipment delays	1 (0.1)	1 (0.1)	6 (0.5)	8 (0.7)
Human resources gaps for public health	1 (0.1)	1 (0.1)	3 (0.3)	5 (0.4)
Inadequate financing or resources for response initiation or rapid resource mobilization	0 (0)	0 (0)	3 (0.3)	3 (0.3)
Limited availability of treatments, countermeasures or personal protective equipment	0 (0)	0 (0)	2 (0.2)	2 (0.2)
Others	1 (0.1)	0 (0)	0 (0)	1 (0.1)
**Other Themes**	**17 (1.5)**	**3 (0.3)**	**14 (1.2)**	**34 (3)**
**Total**	**597 (52.4)**	**234 (20.5)**	**297 (26.1)**	**1128 (98.9)**

* Percentage calculated with number of bottlenecks as the numerator and number of events that reported either bottleneck or enabler (1140) as the denominator

Among the 696 events reporting enablers, coordination mechanisms dominated (449 enablers, 39%), with effective coordination across public health units being the most predominant (243 enablers, 21%). Clinical or health care worker constituted the second major enabling category (225 enablers, 20%), including awareness and clinical suspicion by health workers (200 enablers, 17.5%). Laboratory system enablers (198 enablers, 17%) were additional system strengths. Detection phase enablers were reported in 366 events, notification enablers in 272 events, and response enablers in 396 events. ( Table 5).

**Table 5.** Reported enablers of achieving the 7-1-7 target in Thailand, 1 Jan 2024 − 31 June 2025.

Themes and Categories of Enablers	Detect	Notify	Response	Total
	N (%)	N (%)	N (%)	N (%)
**Coordination**	34 (3)	196 (17.2)	219 (19.2)	449 (39.4)
Effective coordination across public health units and agencies	24 (2.1)	177 (15.5)	42 (3.7)	243 (21.3)
Well-functioning multisectoral/disciplinary response teams	7 (0.6)	7 (0.6)	153 (13.4)	167 (14.6)
Strong One Health information sharing and collaboration	**1 (0.1)**	**7 (0.6)**	**12 (1.1)**	**20 (1.8)**
Strong coordination between public and private sectors	2 (0.2)	5 (0.4)	7 (0.6)	14 (1.2)
Robust response coordination, including incident management and rapid response team capacity	0 (0)	0 (0)	3 (0.3)	3 (0.3)
Effective coordination with neighboring countries	0 (0)	0 (0)	2 (0.2)	2 (0.2)
**Clinical or Health Care Worker**	186 (16.3)	16 (1.4)	23 (2)	225 (19.7)
High awareness and clinical suspicion by health workers	172 (15.1)	12 (1.1)	16 (1.4)	200 (17.5)
Health professionals with adequate training in surveillance and response	7 (0.6)	2 (0.2)	5 (0.4)	14 (1.2)
Sufficient clinical case management capacity	3 (0.3)	0 (0)	2 (0.2)	5 (0.4)
Others	**3 (0.3)**	**1 (0.1)**	**0 (0)**	**4 (0.4)**
Strong clinical surveillance focal point with adequate capacity	1 (0.1)	1 (0.1)	0 (0)	2 (0.2)
**Laboratory**	77 (6.8)	10 (0.9)	111 (9.7)	198 (17.4)
Adequate laboratory diagnostic capacity	25 (2.2)	2 (0.2)	38 (3.3)	65 (5.7)
Reliable and timely laboratory reporting	15 (1.3)	4 (0.4)	39 (3.4)	58 (5.1)
Timely specimen collection	**6 (0.5)**	**2 (0.2)**	**11 (1)**	**19 (1.7)**
Fast internal laboratory turnaround time	5 (0.4)	1 (0.1)	12 (1.1)	18 (1.6)
Fast specimen transportation	11 (1)	0 (0)	6 (0.5)	17 (1.5)
Sufficient diagnostic commodities (lab reagents, RDTs, specimen collection kits)	12 (1.1)	0 (0)	5 (0.4)	17 (1.5)
Others	3 (0.3)	1 (0.1)	0 (0)	4 (0.4)
**Patient & Community**	64 (5.6)	3 (0.3)	4 (0.4)	71 (6.2)
Early care-seeking behavior by patients	49 (4.3)	0 (0)	3 (0.3)	52 (4.6)
High sensitivity of community detection systems	6 (0.5)	0 (0)	1 (0.1)	7 (0.6)
Effective risk communications and community engagement	**3 (0.3)**	**3 (0.3)**	**0 (0)**	**6 (0.5)**
Others	4 (0.4)	0 (0)	0 (0)	4 (0.4)
High community knowledge and trust in health systems	2 (0.2)	0 (0)	0 (0)	2 (0.2)
**Data Systems**	22 (1.9)	37 (3.2)	10 (0.9)	69 (6.1)
Reliable electronic surveillance/reporting systems with good network coverage	**12 (1.1)**	**35 (3.1)**	**3 (0.3)**	**50 (4.4)**
Timely and complete surveillance data availability	10 (0.9)	2 (0.2)	7 (0.6)	19 (1.7)
**Planning & Procedures**	5 (0.4)	8 (0.7)	44 (3.9)	57 (5)
Well-defined surveillance and response policies and guidelines	5 (0.4)	2 (0.2)	29 (2.5)	36 (3.2)
Proper implementation of initial risk assessment and event verification procedures	0 (0)	0 (0)	6 (0.5)	6 (0.5)
Comprehensive risk assessments, preparedness, and response plans	0 (0)	0 (0)	6 (0.5)	6 (0.5)
Consistent adherence to event notification procedures	0 (0)	2 (0.2)	2 (0.2)	4 (0.4)
Clear and adequate procedures for event notification	**0 (0)**	**4 (0.4)**	**0 (0)**	**4 (0.4)**
Others	0 (0)	0 (0)	1 (0.1)	1 (0.1)
**Resouces & Procurement**	6 (0.5)	4 (0.4)	33 (2.9)	43 (3.8)
Ready availability of treatments, countermeasures, and personal protective equipment	5 (0.4)	0 (0)	15 (1.3)	20 (1.8)
Sufficient human resources for public health emergencies	**1 (0.1)**	**0 (0)**	**11 (1)**	**12 (1.1)**
Adequate financing and resources for immediate response initiation and rapid resource mobilization	0 (0)	4 (0.4)	3 (0.3)	7 (0.6)
Efficient logistics and supply chains with minimal delays	0 (0)	0 (0)	4 (0.4)	4 (0.4)
**Event Characteristics**	13 (1.1)	0 (0)	2 (0.2)	15 (1.3)
Known, expected, or prioritized pathogens with established protocols	6 (0.5)	0 (0)	2 (0.2)	8 (0.7)
Others	7 (0.6)	0 (0)	0 (0)	7 (0.6)
**Other Themes**	**4 (0.4)**	**5 (0.4)**	**4 (0.4)**	**13 (1.1)**
**Grand Total**	**411 (36.1)**	**279 (24.5)**	**450 (39.5)**	**1140 (100)**

* Percentage calculated with number of enablers as the numerator and number of events that reported either bottleneck or enabler (1140) as the denominator

## Facilitators, challenges and solutions in applying the 7-1-7 metrics

**Figure 4.**
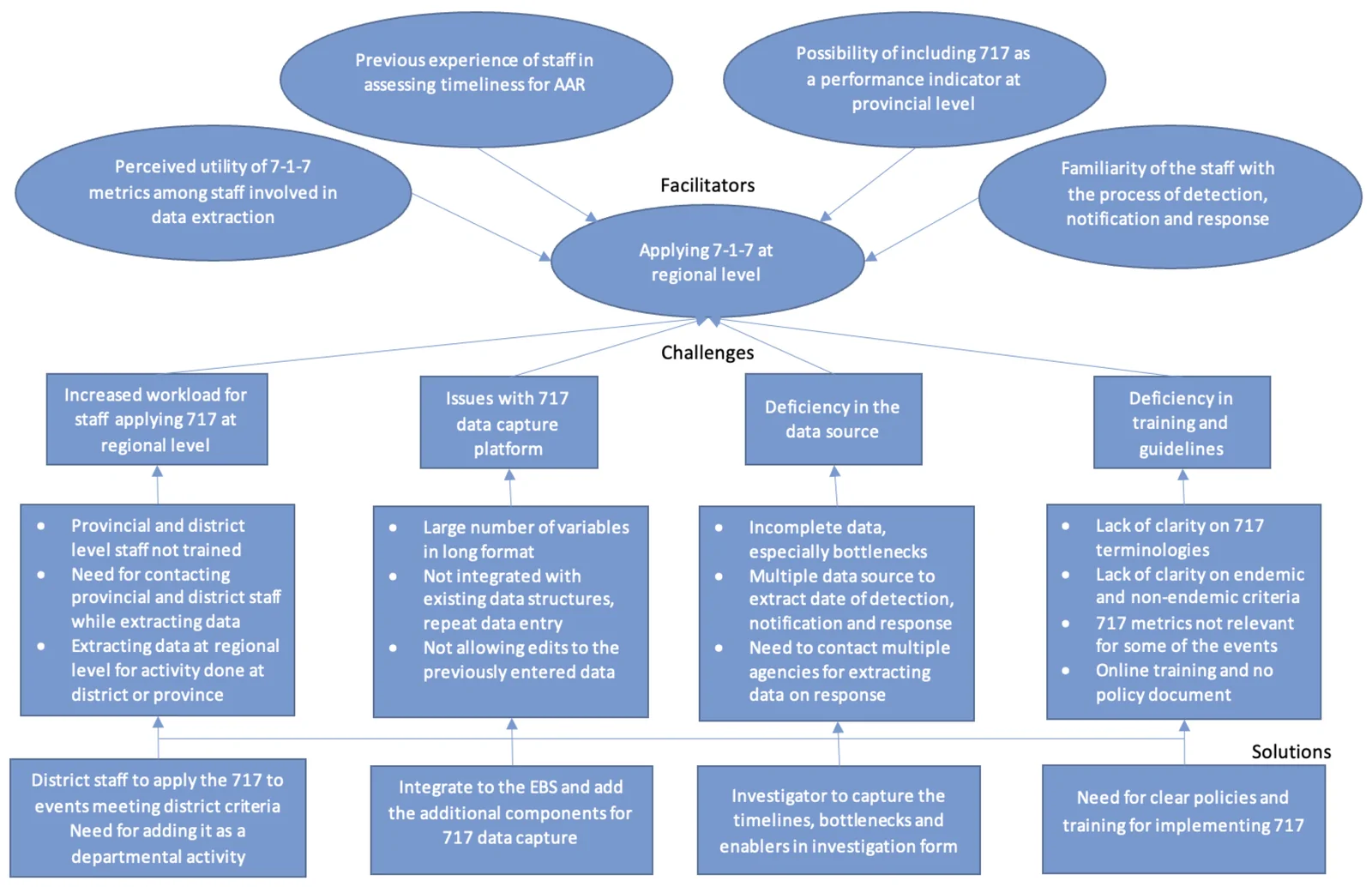
Thematic diagram of the facilitators and challenges in applying the 7-1-7 metrics in Thailand.

The facilitators in applying the metrics, which emerged from the interviews, included the perceived utility of using the metrics, the familiarity of the staff with the processes of detection, notification and response, and previous experiences of assessing timeliness of public health events. Even before the adoption of 7-1-7, different timeliness metrics were being assessed for some communicable diseases like dengue. However, some challenges were faced by data managers, when applying the tool. The absence of a single, standard data source meant that they had to review multiple documents to source the data for entering into EBS-717. In cases where the responses were initiated at the sub-regional level, data entry was still required to be done by the regional staff. When there was data missing in the forms, the sub-regional teams had to be telephoned. Because of these challenges, their workload increased. Additionally, they felt that there was duplication of data entry into EBS as well as EBS-717. Platform challenges like issues with editing previous entries, and difficulties in entering bottlenecks and enablers were reported. Possible solutions to overcome these challenges were proposed, which centred around streamlining and decentralising the process of 7-1-7 application ( Figure 4) ( Table 6).

**Table 6.** Qualitative quotes for facilitators and challenges.

Themes	Categories	Quotes
Facilitators		
Perceived utility of 7-1-7 metrics among staff involved in data extraction		“7-1-7 is a tool for measuring timeliness in detection, reporting, and response. It guides us to look for factors - bottlenecks or enabling factors - that determine whether we can implement each component on time, and helps plan problem-solving. It's a guiding tool that helps us identify these issues.” “When we apply this tool, we can see gaps in some events - what problems caused delays - and use the analysis results to find appropriate measures to overcome these areas. This is better than before when we had no measurement tools. Without detection tools, we'd suggest broad measures rather than specific ones.”
Previous experience of staff in assessing timeliness for after action reviews		“When we investigated diseases, we analysed how quickly each event was detected, why outbreaks became extensive or widespread. This was done for specific events where the region conducted investigations, or before COVID's early outbreak period when we did reviews - like AAR (After Action Review) - discussing whether detection should have been faster. But this was done occasionally when opening Emergency Operation Centres and conducting AARs”
Possibility of including 7-1-7 as a performance indicator at provincial level		“PAOs (Provincial Administrative Organisations) don't adopt all our indicators - that's what they said. So, since they prioritize surveillance systems, if we now introduce 7-1-7, they would be ready to adopt it. This is important for PAOs - just helping their leaders understand. If their leaders understand, their subordinates - not subordinates, but people working in public health areas - will have some of our people, meaning people from the Ministry of Public Health transferred.”
Familiarity of the staff with the process of detection, notification and response		“The concept of 7-1-7 is something we were already doing. What we were already doing includes surveillance systems, outbreak detection in event-based or current EBS systems, which we were already using. But we didn't see clearly how it was grouped. Now it's grouped to provide more clarity on what we do. Same with response - we were already responding, but this made it clearer that it should be within 7 days. What we were already doing is when there are events or incidents in areas - besides what we already know - first, we notify areas and all relevant parties. We notify teams, whether investigation teams or our own SAT teams, and local area teams too. This 7-1-7 grouped things to make what do clearer, followed by response activities. For response, we already respond - we don't wait 7 days.”
Challenges		
Increased workload for staff applying 7-1-7 at the regional level	Provincial and district level staff not trained	“We haven't communicated to provincial or district levels about what we're doing. It's still within ODPC (Office of Disease Prevention and Control) circles and people who've been trained for preliminary assessment.”
	Need for contacting provincial and district staff while extracting data	“I have problems with some events where we follow up with provinces but don't get information, or some events we follow up on but they haven’t entered it into the Event-based program, so we can't continue inputting.” “If we find events that should be in 7-1-7 but can't be inputted due to incomplete data, our team needs to follow up.”
	Extracting data at regional level for all activities	“Like if district-level response is assessed by the district itself, it's clear because data is in their hands. But like what we're doing now - having regions assess provincial or district responses - we lack data, making it difficult and time-consuming for each event, and we're unsure of accuracy.”
Issues with 7-1-7 data capture platform	Large number of variables in long format	“Platform issues - when entering data, having to separate different sections. It looks overwhelming when entering. If you forget to enter something, you need to scroll up to find it, which can be confusing.” “Yes, so when accessing specific sections, it's easier to select. The current long format makes it slow to find missed entries.”
	Not integrated with existing data structures, repeat data entry	“Currently, we still rely on the original system. We use the original criteria first, then input the data retrospectively. We haven't fully implemented 7-1-7 yet - we haven't integrated 7-1-7 into our investigation forms or required paperwork. We're still doing things the old way and then analysing how it fits with 7-1-7 during data entry”
	Not allowing edits to the previously entered data	“Probably system stability - after entering data, when we return to edit, the data sometimes disappears. Sometimes we have to sit and re-enter everything.”
Deficiency in the data source	Incomplete data, especially bottlenecks	“It might be somewhat difficult because when they send us information, it's often not very complete.” “Sometimes patients come for treatment late, but reports only say they came for treatment without explaining why they were delayed - lacking clear information for factor analysis.”
	Multiple data sources to extract date of detection, notification and response	“The key issue is data - various data sources we use for assessment. If accessing this data was easy, then processes we're already doing become manageable”
	Need to contact multiple agencies for extracting	“In the past, when opening EOCs, we organized by functional groups. SAT handled detection issues, operations groups handled response functions. We usually divided by functional groups, but another approach was to divide by EOC strategic objectives – each group looked after whether each strategy was addressed.”
Deficiency in training and guidelines	Lack of clarity on 7-1-7 terminologies	“Some terminologies are confusing. During meetings, we gave feedback asking for definitions of each term, like "emergence date" or "detection date." Sometimes it's unclear - for example, with patient clusters, if one person gets sick on one day and comes to the hospital on another day, do we use the hospital visit date or the onset date for "detection date"? There are many terms we still don't understand.”
	Lack of clarity on endemic and non-endemic criteria	“Mainly analysing whether events meet the endemic or epidemic criteria - the manual isn't clear enough. We can't understand some events to determine if our entries are correct.”
	7-1-7 metrics not relevant for some of the events	“From what I hear, diseases unsuitable for 717 would be: 1) diseases with long onset periods or incubation periods like leprosy or XDRTB requiring lab time or expertise for diagnosis, and 2) for drowning, it's not communicable and detection might not be answerable since no one witnesses events.”
	Online training and no policy document	“I'd like more training, preferably on-site rather than online. Online is accessible but might not cover all issues clearly. I'd like to invite management to see 7-1-7's importance and how it could connect and integrate with existing work. I'm not sure if 7-1-7 wants us to input new cases immediately or just work with the database from where we extract first.”
Solutions		
District staff to apply 7-1-7 to events meeting district criteria		“I think we should convey information to provincial and district level and let them discuss further about what 7-1-7 is and how they can help regional centres, or how provinces and districts might need to use 7-1-7 themselves for their own quality improvement. When we perform assessments, we encounter problems that provinces and districts don't investigate on time. So, I think this could be a tool to expand if we see it helps with investigation quality and standards.” “But it can be expanded for areas (districts) to implement - I think it's possible. They could report more complete information than regional centres because they're closer to the areas.”
Need for adding it as a departmental activity		“Some people might be interested while others aren't. It needs to become a real departmental policy rather than just optional minor work.”
Integrate it with the event-based surveillance system (EBS) and add the additional components for 7-1-7 data capture		“To reach that point, we might need to create linkages with surveillance systems. For example, if we record data about current cases in EBS, whether events meet the investigation criteria or not, if we can link them using event codes, then add specific 7-1-7 questions about implementation steps - whether these steps were implemented, whether areas implemented them - this would create work integration.”
Investigator to capture the timelines, bottlenecks and enablers in the investigation form		“At least investigation forms used should include timeline, date and factor components so we don't need to search for them.”
Need for clear policies and training for implementing 7-1-7		“I think we need clear policies - whether it's departmental or divisional level. Make it prominent that this year the department is pushing 7-1-7 for regional centres or driving the epidemiology division to implement it provincially because it will make work easier.”

## Discussion

This country-wide, comprehensive evaluation of 1,435 public health events against the 7-1-7 target is the largest evaluation done globally till date. The finding that half of all events met the 7-1-7 target represents a substantial achievement for a middle-income country implementing global health security standards. Achieving detection within seven days in 85% events suggests that Thailand's surveillance infrastructure has matured considerably since the establishment of its event-based surveillance system. However, when examined by disease category, notification of DCDs requires further improvement, as timely notification is mandated for all such diseases, but it was found that only about 95% of cases were notified within one day. Additionally, when considering settings, control measures in hotels were delayed. The most common bottlenecks occurred within patients and communities, which hampered the effectiveness of disease control efforts.

Thailand demonstrated remarkable early response performance with a median response time of 1 day. This superior performance stems from Thailand's robust legislative framework, particularly the Communicable Disease Act 2015 ^11^, which mandates establishment of a CDCU in every district, with specific notification and response requirements. The act facilitated the formation of CDCUs to conduct disease investigations with trained professionals, creating systematic outbreak response capabilities. Additionally, Thailand's standardized SRRT protocols incorporate timeliness as a core performance indicator, ensuring consistent rapid response across all levels ^14^. These comprehensive guidelines enabled Thailand to respond in a timely manner and represent four decades of investment in surveillance and preparedness integrated into district health systems.

The Thai Communicable Diseases Act, 2015, mandates that all DCDs must be reported to the DDC within 3 hours of detection, aligned with the next-1 of the 7-1-7 metrics. However, in this evaluation, 9% of events were reported later than the one-day timeframe; most of them were XDR-TB cases. The duration for notification from detection was an anomaly introduced due to processes involved in the diagnosis of XDR-TB. All XDR-TB patients are initially diagnosed to have TB using sputum smear microscopy or Xpert assays. The date of diagnosis of TB is considered as the date of detection. The samples from diagnosed TB patients are subjected to bacterial culture confirmation to diagnose XDR-TB, which takes 3-12 weeks for detecting the resistance patterns ^15, 16^. Because notification happens only after the culture results are confirmed as XDR-TB, the time from detection to notification of XDR-TB will be usually longer than seven days. This anomaly can be overcome by calculating the detection timeliness as the difference between the date of symptom emergence and the date of detection of TB and calculating the notification timeliness as the difference between the date of culture confirmation and the date of notification of XDR-TB.

Vaccine-preventable diseases demonstrated particular challenges in achieving the 7-1-7 target, with response completion notably slower than other disease clusters. Majority of these events were Pertussis, Diphtheria and Measles. The bottleneck analysis reveals that "Patient & Community" factors represented the largest category, followed by "Clinical or Health Care Worker" issues, highlighting critical gaps in community awareness and healthcare-seeking behaviour. Furthermore, a delay in laboratory confirmation, like confirming the presence of toxin-producing Diphtheria, generally takes a long turn-around time ^17^. Therefore, improving performance against the 7-1-7 framework is essential for maintaining elimination goals and preventing outbreaks in susceptible populations.

The poor results in hotels, where no events met the 7-1-7 target, and detection was much lower than other settings, are mostly due to Legionnaires’ disease. This matches previous European reports, which also found delays in detecting Legionnaires’ disease ^18^. Delayed detection could be because doctors do not suspect atypical pneumonia at first, but rather test for common infections ^19^. Only when these come negative is Legionella tested for, leading to delayed diagnosis. Tourists may also leave the area before the cause of pneumonia has been identified, thus further delaying detection ^20^. To reduce deaths and prevent outbreaks in travellers, hotels need regular water testing, cleaning, and better disease surveillance ^21^. Also, strengthening international coordination through standardized reporting approaches to detect and respond to clusters of travel-associated Legionnaires' disease would improve outbreak control in hotels ^22^. In contrast to hotels, 100% of events in religious settings were detected in time. Most of these events were contagious respiratory infections like influenza and Covid-19, for which there was a high index of suspicion and easily available, universally affordable rapid test kits to enable timely detection.

Bottlenecks identified for detection included significant delays in care-seeking behaviour, and insufficient community knowledge and trust in healthcare services ^23^. These barriers represent critical intervention points, as research demonstrates that trust in healthcare systems has an impact on care utilization similar in magnitude to established factors such as income and education ^24– 26^. In Thailand's context, factors facilitating healthcare access are multifaceted and include both systemic improvements in health service delivery and community-level interventions. Additionally, national surveys found that only 6% of the Thai population had high levels of health literacy, while half the population had low levels ^25^. Thus, prioritising health literacy interventions is crucial to improve performance against the timeliness metrics.

Through the interviews, challenges experienced by the staff applying the 7-1-7 metrics were brought forth. It is likely that the difficulties and burdens faced in extracting data from multiple sources before entering it into EBS-717, as well as the duplication of work, contributed to 45% of events having incomplete data. Users also perceived challenges in entering bottlenecks and enablers in the current database, which would have further increased the time taken for data entry, and may have led to incomplete reporting. Additionally, some interviewees argued that certain diseases, such as tuberculosis, should be excluded from the metrics. Others felt that certain events should be detected in much less than seven days, and notified within a few hours of detection; therefore, the 7-1-7 target was irrelevant for such events. Multiple solutions to overcome the challenges emerged, such as adopting 7-1-7 as a departmental activity, extending it to the district and provincial levels, and forming clear policies for implementing the metrics.

## Thailand’s next steps

To overcome the crucial challenges reported and to optimize the application of the 7-1-7 metrics in Thailand, the following steps are proposed: (1) better integration of the EBS-717 database into the national EBS, with automatic filling of all possible fields on entering the event ID; (2) public health staff at all levels able to report data into the system, and access the information to increase utilisation; (3) establish disease prioritization and contextualize the criteria to define which diseases/events the 7-1-7 metrics should be applied to; (4) the 7-1-7 framework should be integrated into the NAPHS, with policies issued by the MOPH or the DDC, to ensure its adoption into routine workflows. The NAPH should also incorporate identified bottlenecks and enablers, to support the consistent achievement of the 7-1-7 target.

## Study strengths and limitations

This study’s strengths include its national scope, comprehensive data collection with inclusion of all events, and its mixed-methods design. The 18-month observation period with 1415 public health events provides valuable baselines of the timeliness of detection, notification, and early response for each setting and disease. Additionally, the bottleneck and enabler taxonomy helped us identify and prioritize opportunities to enhance Thailand’s system performance. However, several limitations should be considered when interpreting the findings. First, data completeness varied considerably across the 7-1-7 components, 88% for detection, 83% for notification, and 59% for response, potentially biasing the performance estimates. Second, although bottlenecks and enablers were identified, the interventions or strategies implemented to address these challenges have not been reported. Therefore, future research should focus on longitudinal assessments of the progress, effectiveness, and sustainability of such actions after these gaps are addressed.

## Conclusion

Between January 2024 and June 2025, around half of all the assessed events met the 7-1-7 target in Thailand. Performance varied by disease and setting, with delays observed in reporting drug-resistant tuberculosis, and in detecting outbreaks in hotels. Key bottlenecks identified were in patient care-seeking behaviour, community awareness, and inter-agency coordination, while effective coordination and laboratory capacities served as prevalent enablers. The adoption of the 7-1-7 framework led to improvements across multiple levels, including a trained workforce, data systems development, and One Health integration. However, efforts are required to improve data completeness and evaluate the interventions which address the identified bottlenecks. Next steps for Thailand include improving data integration, prioritizing diseases for surveillance, and decentralizing the application of the metrics. Incorporating the identified bottlenecks into Thailand’s NAPHS will be critical to improve outbreak preparedness and response, ultimately contributing to improved public health outcomes.

## Data Availability

The datasets generated and analyzed during this study contain sensitive public health surveillance data that include potentially identifiable health event information and geographic details. In accordance with the ethical approval granted by the Institute for the Development of Human Research Protections (IHRP), Thailand (approval number: IHRP No.45-2568, dated 28 April 2025), and Thailand's data protection regulations, these data cannot be made publicly available to maintain the security of Thailand's disease surveillance systems. The data for this study can be obtained upon reasonable request by submitting a formal data access request to the Department of Disease Control ( ddc.moph@ddc.mail.go.th). Requests will be reviewed by the study team and the Department of Disease Control under the following conditions: A detailed research proposal demonstrating legitimate scientific objectives Approval from the applicant's institutional ethics committee A signed data sharing agreement ensuring data security and confidentiality Agreement not to attempt re-identification of individuals or locations Acknowledgment of the data source in any resulting publication Figshare: Implementation of 7-1-7 for improving timeliness in outbreak detection and response in Thailand: A mixed-methods study on challenges in application and achievement of targets in 2024–25 All materials that can be made publicly available have been deposited as supplementary files in Figshare under the DOI: https://doi.org/10.6084/m9.figshare.30926906 ^27^. Extended data includes: Supplementary 1 Thailand health events that meet the national criteria for health threats Supplementary 2 Qualitative questionnaire to assess the enablers and challenge in applying 7-1-7 framework Supplementary 3 Research Participant Information Supplementary 4 Interview Guide for Executive Participants Supplementary 5 Interview Guide for Practitioner Participants Supplementary 6 Informed Consent Form Supplementary 7 Timeliness and proportion of events achieving 7-1-7 target by disease, Jan 2024–June 2025 Data are available under the terms of the Creative Commons Attribution 4.0 International license (CC-BY 4.0).
