# Supplementary material for: Implementation of 7-1-7 for improving timeliness in outbreak detection and response in Thailand: A mixed-methods study on challenges in application and achievement of targets in 2024-25
Source: Wellcome Open Res. 2026 Feb 12;11:108. [Version 1] doi: 10.12688/wellcomeopenres.25087.1 (PMC13583354; doi:10.12688/wellcomeopenres.25087.1)
Supplement: Peer review history

# Reviewer report: Reviewer response for version 1

**DOI:** 10.21956/wellcomeopenres.27657.r165850

**Published:** 2026-09-16

**Authors:** Arkalgud Ramaprasad

**Affiliations:** 1 University of Illinois at Chicago, Chicago, Illinois, USA

The focus of the paper is on implementation of 7-1-7 for improving timeliness in outbreak detection and response in Thailand. In studying the challenges of application of the method and achievement of targets, it foucses symmetrically on the desired norm (7-1-7), enablers (drivers), and bottlenecks (barriers). Its approch is balanced. The study is thorough, detailed, and clear in its presentation. The subject of the study is important and the lessons drawn from it are applicable to other countries with similar public health problems.

 The authors may want to consider recasting the conclusions and recommendations from the study using a feedback and learning framework. In this day of machine learning and data science, they want to project the possibility of reconceptualizing publich health surveillance as a flexible, adaptive system with proactive/quick learning instead of as a fixed, non-adaptive system with reactive/slow learning. Such a reconceptualization would go beyond the incremental next steps described on page 19.

 Consider for example the Disease Surveillance Systems (DSS) portrayed in Figure 2 with feedback loops from EBS to DDS and EBS to Laboratories respectively. These feedback loops could be digitized with machine learning with human supervision to reduce the lag intrduced by  human actors alone. They could also reduce the errors/oversights in the systems through timely indication and correction.  Even if the present DSS data are incomplete and error-prone, a suitable feedback and learning system can help make them more complete and error-free. A similar feedback and learning system can be conceptualized from the lower levels of the hierarchy of the public health system shown in Figure 1 to the upper levels.

 The conclusion of the paper suggests that “future work should focus on strengthening data integration and formulating disease specific guidelines.” The authors may want to be more ambitious based on their study and present a vision of digitalized public health surveillance for Thailand that starts with proactive, primordial prevention based on learning from past data instead of on reactive, prevention based on analysis of present data.

Is the work clearly and accurately presented and does it cite the current literature?

Yes

If applicable, is the statistical analysis and its interpretation appropriate?

Yes

Are all the source data underlying the results available to ensure full reproducibility?

Yes

Is the study design appropriate and is the work technically sound?

Yes

Are the conclusions drawn adequately supported by the results?

Yes

Are sufficient details of methods and analysis provided to allow replication by others?

Yes

Reviewer Expertise:

Ontological meta-analysis and synthesis

I confirm that I have read this submission and believe that I have an appropriate level of expertise to confirm that it is of an acceptable scientific standard.

## References

[1] : On the definition of feedback. *Behavioral Science* .1983;28(1) : 10.1002/bs.38302801034-13 10.1002/bs.3830280103

[2] Ramaprasad, A., & Syn, T. (2026, March 23). Colloquial-Feedback or Cybernetic-Feedback for the NIH? [Substack newsletter]. Arkalgud Ramaprasad. https://ontologizer.substack.com/p/colloquial-feedback-or-cybernetic.

# Reviewer report: Reviewer response for version 1

**DOI:** 10.21956/wellcomeopenres.27657.r154322

**Published:** 2026-06-19

**Authors:** Steven Riley

**Affiliations:** 1 Imperial College London, London, England, UK

In this paper, public health colleagues from Thailand report a statistical analysis of the countries implementation of the 7-1-7 outbreak objectives in which every outbreak is to be detected within 7 days, reported within 1 day and responded to within a further 7 days. They evidence that 50% of outbreaks meet that target but that some types in particular tend not to be reported quickly enough. They highlight the need for better data integration. 

 This rich and well-presented report that will be of use to many health security colleagues around the world.

 Additional comments

 Page 1, column 1, line +4: Who proposed the 7-1-7 target?

 2,1,+ 4: What made these dangerous? is there a more common term.

 2,1,+5: how do the last 3 percentages relate to the 50? Is there a reporting element to the 50%. Need to be clear about outbreak for which there is complete data versus those where only partial data are available.

 4,1, +20: Please make it clear here that for non-endemic disease emergenc is the day of onset. This is clear in a table much further down in the article.

 4,1,-5: Please cite the alliance website.

 4,1,-2: Please give a brief introduction to tie regions before calling our region 2. 

 5,2,+8: What exactly is the Outbreak response system at this point. Who is it run by?

 5,2,-14: Noting the earlier comment about dangerous communcable diseases and the acronym DCD. Define these earlier before using if possible. 

 Table 1: Noting that the detection clock starts from first symptoms in a non-endemic pathogen. Please mention this earlier.

 8,2,+15: This paragraph was difficult to follow. I couldn’t tell which % related to which subset. Table 2 makes it more clear but is difficult to read. I suggest the page is rotated in the pdf if possible. But the main point is to be really clear in the paragraph what is the numerator and denominator for each percentage… “Of those events of type x for which complete data were available we found…”

 Figure 3. Bottlenecks and enables need a brief definition in the figure legend.

 15,2,+12: suggest that XDR-TB is treated differently overall to highlight that there is some unresolved risk there. Onset day is still the right starting point, but current processes mean it cannot be compliant with the 717 system. Keeping that explicit will ensure that finding better solutions for routine TB typing remain a priority.

 18,2,+1: which types of outbreaks are being talked about here and what might be a reasonable time for care seeking behaviour for which symptoms. I’d be interested in a few examples here: is it fever then community pneumonia? How long is it taking? What could we reasonably expect?

 19,1,+22: On next steps, I’d also emphasise tracking trends. The absolute targets are good but are a bit arbitrary and difficult to compare between different information systems. But year on year, comparing regions against each other and looking at different cluster types, it might be possible to see genuine improvements.

 Data availability: Is there a safe, anonymised row level data set that might be useful to other researchers. Perhaps adding noise to the initial date for a record would make it unidentifiable but would preserve most of the information in the data.

Is the work clearly and accurately presented and does it cite the current literature?

Yes

If applicable, is the statistical analysis and its interpretation appropriate?

Yes

Are all the source data underlying the results available to ensure full reproducibility?

Yes

Is the study design appropriate and is the work technically sound?

Yes

Are the conclusions drawn adequately supported by the results?

Yes

Are sufficient details of methods and analysis provided to allow replication by others?

Yes

Reviewer Expertise:

Infectious disease and public health

I confirm that I have read this submission and believe that I have an appropriate level of expertise to confirm that it is of an acceptable scientific standard.
